# Clinical Effectiveness and Safety of Electrochemotherapy (ECT) in the Treatment of Locally Advanced Melanoma: A Systematic Review

**DOI:** 10.7759/cureus.98402

**Published:** 2025-12-03

**Authors:** Daniel J Vickars, Anirban Mandal

**Affiliations:** 1 Hepatology, University Hospital Southampton NHS Foundation Trust, Southampton, GBR; 2 Plastic Surgery, Whiston Hospital, Mersey and West Lancashire Teaching Hospitals NHS Trust, Prescot, GBR

**Keywords:** cancer immunotherapy, cutaneous oncology, electrochemotherapy, malignant melanoma metastasis, melanoma and immunotherapy, metastatic skin cancer, stage iii melanoma

## Abstract

Electrochemotherapy (ECT) is a locoregional cancer treatment primarily used in the palliative management of cutaneous metastases from malignant melanoma. However, its clinical effectiveness, safety, and cost-effectiveness in this setting are poorly defined. The aim of this systematic review is to assess the clinical effectiveness, safety, quality of life (QoL) outcomes, and cost-effectiveness of ECT in the treatment of locally advanced stage III/IV melanoma with in-transit metastasis or loco-regional recurrence. A literature search of MEDLINE and Embase from 2005 to 2024 was performed using the keywords “electrochemotherapy” and “skin cancer”. After duplicate removal, 708 studies were identified, 107 English-language papers from 2020 to 2024 were screened, with 47 full texts reviewed. Ten studies met the inclusion criteria and were included for review. Data extraction and reporting adhered to the Preferred Reporting Items for Systematic Reviews and Meta-Analyses (PRISMA) Guidelines. ECT achieved complete response (CR) rates of 24-85.7%, overall response rates (ORRs) of 63-100%, and one-year overall survival of 60.4-92.6%. ECT was well tolerated with minimal systemic toxicity and minor adverse effects. QoL was maintained post-treatment, and cost-effectiveness analysis suggested economic viability in particular patient cohorts. ECT demonstrated improved tumour control and survival outcomes when combined with systemic immunotherapy. ECT is a safe, well-tolerated, and clinically effective treatment for stage III/IV metastatic melanoma, providing a valuable alternative for patients unsuitable for surgery or systemic chemotherapy. Further prospective RCTs are needed to establish its long-term role in melanoma care.

## Introduction and background

Melanoma is an aggressive cutaneous malignancy accounting for approximately 4% of skin cancer diagnoses but responsible for approximately 80% of skin cancer-related deaths globally [1]. The incidence of melanoma continues to rise globally, which is largely attributed to increased UV exposure and an aging population. In the UK, around 17,500 new cases of melanoma are diagnosed annually, with approximately 2,300 deaths [2]. Prognosis is stage-dependent, with stage I/II melanomas treated with curative intent through surgical excision, achieving five-year survival rates of approximately 90% [3]. However, patients with metastatic melanoma (stage III/IV) face significantly poorer prognosis, with a median survival time of eight to nine months and an estimated three-year survival of 10-15% [4]. Treatment options for stage III/IV melanoma include surgical excision and systemic chemotherapy, as well as novel immunotherapies and immune checkpoint inhibitors, such as nivolumab or pembrolizumab [5,6]. In patients with loco-regionally advanced disease, the lesion may be unresectable due to tumour burden, anatomical complexity, or patient frailty, and in such cases, effective non-surgical treatments that preserve function and maintain quality of life (QoL) are essential. 

Electrochemotherapy (ECT) has emerged as a locally ablative therapy that can be used for the treatment of cutaneous malignancies and metastases [7-9]. ECT combines the delivery of chemotherapeutic agents, most commonly bleomycin, with high-voltage electric pulses that transiently permeabilise tumour cell membranes to enhance intracellular drug uptake and cytotoxicity [10]. Originally developed for palliative management of superficial metastases, ECT has demonstrated high localised tumour response rates, excellent tolerability and the potential to downsize the lesion before surgery [11]. In 2006, the European Standard for Operating Procedure for Electrochemotherapy (ESOPE) was established to provide standard operating procedures for ECT [12]. This helped to broaden the applications of ECT, which now include the treatment of recurrent melanoma and superficial metastases. Despite increasing clinical interest, the overall effectiveness of ECT in this specific setting remains incompletely defined. This systematic review aims to review the existing gap in the current literature on ECT as a treatment for stage III/IV melanoma with in-transit metastases or loco-regional recurrent melanoma, with a focus on clinical effectiveness, safety, QoL outcomes, and economic viability.

## Review

Methods

**Figure 1:**
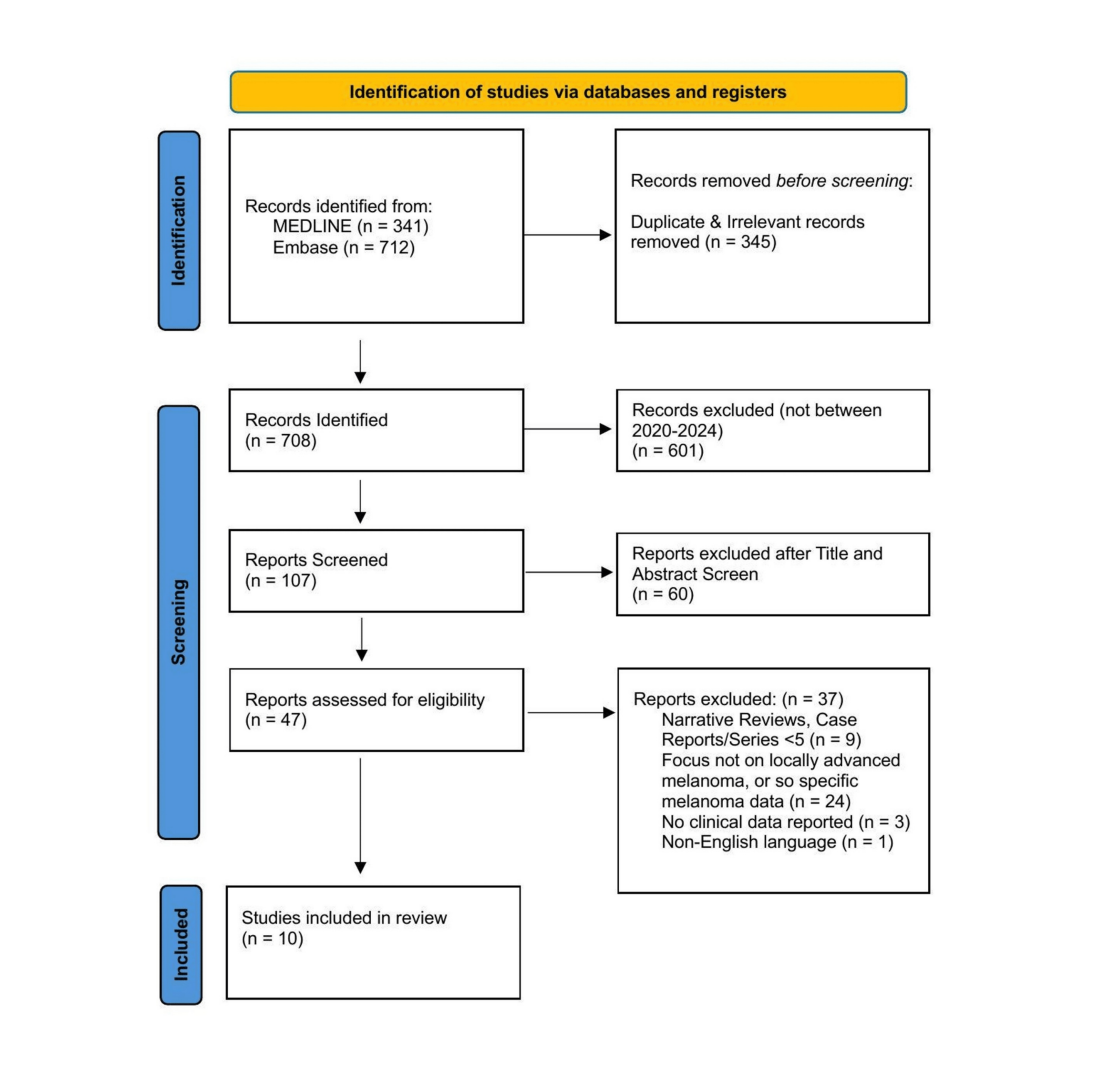
Preferred Reporting Items for Systematic Reviews and Meta-Analyses (PRISMA) flow diagram

An electronic literature search was performed in MEDLINE and Embase of studies published from January 2005 to April 2024. The search strategy used the key terms "electrochemotherapy" AND "skin cancer”. After duplicate removal, 708 records were identified, and the search results were restricted to English-language papers from 2020 to 2024. A total of 107 articles were identified and underwent title and abstract screening, which identified 47 papers to which our predefined inclusion and exclusion criteria were applied. Ultimately, 10 studies met the criteria and were included for final analysis. The full selection process is detailed in the PRISMA flow diagram (Figure 1), and the full list of search terms and result counts for MEDLINE and Embase are provided in Appendix A. Studies were independently screened and reviewed by a single investigator according to the predefined inclusion and exclusion criteria, with no formal risk-of-bias assessment performed. This systematic review adhered to the Preferred Reporting Items for Systematic Reviews and Meta-Analyses (PRISMA) 2020 guidelines. No prospective protocol registration was undertaken.

Studies were included if they 1) involved human participants with reported clinical outcome data; 2) were published in English between January 2020 and April 2024; 3) focused specifically on ECT for the treatment of malignant melanoma, including primary and local metastases; 4) reported measurable clinical outcomes for melanoma including tumour response, survival times, adverse events, QoL or cost-effectiveness; and 5) were prospective or retrospective cohort studies, registry studies, systematic reviews, meta-analyses, and economic evaluations.

Studies were excluded if they 1) did not report data specific to melanoma; 2) focused on distant metastasis or non-melanoma skin cancers; Were case reports, case series with <5 patients, narrative reviews, editorials, or opinion pieces without clinical outcome data; 3) were non-English publications; and 4) involved non-human, in vitro, or preclinical models.

Data extraction used a standardised data collection form, and extracted variables included study design, sample size, ECT protocol, clinical outcomes, safety/adverse events, and any other additional clinical outcomes. The extracted data are presented in Appendix B. Due to the variability in study design, population characteristics, treatment protocols, and outcome measures, a formal meta-analysis was not feasible. Instead, a narrative synthesis was conducted. Results were categorised by outcome domain: clinical effectiveness of ECT, safety, QoL outcomes, cost-effectiveness, and additional observations. Studies were qualitatively compared to identify patterns, divergences, and gaps in the evidence relevant to the clinical use of ECT in melanoma. No formal risk of bias or quality appraisal was performed in this review. 

Results

Study Characteristics

The included studies comprised six retrospective or prospective clinical cohort studies and case series [13-18], two systematic reviews [19,20], one meta-analysis [21], and one cost-effectiveness analysis using a Markov model [22]. Sample sizes ranged across the included studies and included both single-centre and multi-centre cohorts across European institutions. All studies utilised bleomycin-based ECT protocols, administered intravenously or intratumorally, in accordance with the ESOPE. 

Patient populations primarily consisted of stage III or loco-regional stage IV melanoma, with lesions involving the skin, subcutaneous tissue, or in-transit metastases. Reported outcomes included complete and overall tumour response rates, progression-free survival and overall survival, adverse events, QoL measures, and cost-effectiveness indicators. All results were patient-based unless specified. Significant heterogeneity in study design and reported outcomes, which limits direct comparability across studies. 

Clinical Effectiveness of ECT

ECT demonstrated consistently high rates of local tumour control across the included studies, with complete response rates (CRRs) ranging from 24% to 85.7% and overall response rates (ORRs) between 63% and 100%. One-year overall survival ranged from 60.4% to 92.6%, and one-year local progression-free survival (LPFS) was 55.5-86%. A summary of the reported CRR and ORR across each study is included in Table 1. 

**Table 1:** Summary of reported complete responses (CRRs) and overall response rates (ORRs)

Study	CRR (%)	ORR (%)
Campana et al. (2024) [15]	24% (initial), 39% (best)	63% (initial), 74% (best)
Ahmad et al. (2024) [19]	15–100% (varies by stage/combination)	40–100% (varies by stage/combination)
Zdzienicki et al. (2024) [17]	45%	80%
Ferioli et al. (2023) [20]	53.5% (lesion-based), 35.7% (patient-based)	77% (lesion-based), 80.6% (patient-based)
Lyons et al. (2023) [18]	85.7% at 3 months	100% at 3 months
Campana et al. (2022) [14]	47%	80%
Campana et al. (2021) [13]	48.9% (combo), 43.9% (ECT alone)	77.8% (combo), 80.5% (ECT alone)
Borgognoni et al. (2020) [16]	59.1% (per patient), 58.6% (per lesion)	88.6% (per patient), 82% (per lesion)
Petrelli et al. (2020) [21]	48% (pooled)	77.6% (pooled)
Pirc et al. (2020) [22]	Modelled: assumed 61% clinical benefit	Modelled: assumed 61% clinical benefit

In a prospective cohort study of 44 patients with stage III/IV metastatic melanoma, Borgognoni et al. reported a patient-based CRR of 59.1% and ORR of 88.6%, with a per-lesion CRR of 58.6% and ORR of 82% [16]. Smaller lesions (<3 cm) demonstrated superior responses compared with larger ones (>3 cm) (CRR 61.3% vs. 31.8%) [16]. Using prospective data from the InspECT registry, Campana et al. (2022) observed a CRR of 47% and ORR of 80%, with durable local control at one, two, and three years (78%, 68%, and 62%, respectively). Stable disease and progressive disease occurred in 13% and 5% of patients, and baseline QoL was identified as an independent predictor of response [14]. Lyons et al. (2023) reported that high-frequency electroporation (HF-EP) with bleomycin achieved a CRR of 85.7% and ORR of 100% at 12 weeks in 42 melanoma lesions [18].

Further evidence of durable locoregional control was reported by Campana et al. (2024) in patients with limb-confined in-transit melanoma metastases, where a single ECT session achieved an initial CRR of 24% and ORR of 63% [15]. Following retreatment, outcomes improved to CRR 39% and ORR 74%. Favourable prognostic factors included treatment-naïve status, tumour size <3 cm, <10 metastases, and use of hexagonal electrodes. Median LPFS was 2.75 years, and complete responders had significantly longer OS (6.4 vs. 5.2 years, p = 0.001) [15].

Long-term data from Zdzienicki et al. in 88 patients with unresectable locoregional recurrent melanoma demonstrated a CRR of 45%, PR of 35%, and ORR of 80%, with one-, three-, and five-year OS rates of 70%, 51%, and 28.5%, respectively [17]. Median OS was 37 months, and median PFS was five months. Notably, patients who received systemic immunotherapy after ECT had significantly improved survival compared with those who did not (one-, three-, and five-year OS: 92.6%, 75.7%, and 47% vs. 62%, 42%, and 21%) [17].

The synergistic potential of ECT with immunotherapy was also demonstrated across other studies. Campana et al. (2021) found that combined ECT and pembrolizumab therapy achieved superior local and systemic responses compared with either treatment alone (CRR 48.9%, ORR 77.8%), with a 1-year LPFS of 86% and OS of 88%, compared to 51% and 64% with pembrolizumab monotherapy [13]. Similarly, Ahmad et al. (2024) reported enhanced response rates when ECT was combined with adjuvant therapies such as immunotherapy or gene electroporation, achieving CRRs of 80-100% for Stage III and ORRs up to 77.8% for Stage III/IV disease [19]. A summary of the studies evaluating ECT combined with immunotherapy is presented in Table 2.

**Table 2:** Summary of reported outcomes of ECT and immunotherapy ECT: electrochemotherapy, CRR: complete response rate, ORR: overall response rate, OS: overall survival

Study	Combination strategy	Outcomes
Campana et al. (2021) [13]	ECT + pembrolizumab	CRR: 48.9%, ORR: 77.8%, one-year OS: 88%, superior to mono-therapy arms
Zdzienicki et al. (2024) [17]	ECT followed by systemic immunotherapy	Five-year OS: 47% with immunotherapy vs. 21% without
Ahmad et al. (2024) [19]	ECT + immunotherapy/gene therapy (varied agents)	CRR: up to 100% in some subgroups, notably higher with immunotherapy

Systematic reviews further corroborate these clinical findings. Ferioli et al. reported pooled lesion-based CRR and ORR rates of 53.5% and 77%, and per-patient CRR and ORR rates of 35.7% and 80.6%, with one- and two-year local control rates of 80% and 72-87%, and one-year OS of 67-86.2% [20]. Petrelli et al. observed similar pooled CRR (48%) and ORR (77.6%) values, noting slightly higher responses with intratumoural versus intravenous bleomycin administration (81.9% vs. 69.2%) [21]. Ahmad et al. (2024) additionally demonstrated that ECT outperformed chemotherapy alone (CRR 72-74% vs. 13-26%; ORR 78% vs. 38%) [19]. Pirc et al. [22], while not reporting clinical outcomes directly, estimated a clinical benefit rate of 61% in their cost-effectiveness model, supporting ECT’s value as a repeatable and economically viable treatment option.

*Safety* 

ECT was consistently reported as a safe and well-tolerated treatment across all included studies, with adverse events predominantly local, self-limiting, and rarely severe. A summary of the adverse events in each study is displayed in Table 3.

**Table 3:** Summary of reported adverse events

Study	Adverse events
Campana et al. (2024) [15]	40% skin toxicity, 15% grade ≥3 ulceration, more with combo therapy
Ahmad et al. (2024) [19]	Minimal toxicity, even with combination therapies
Zdzienicki et al. (2024) [17]	Not reported, but no concerns raised
Ferioli et al. (2023) [20]	Ulceration, necrosis (up to 41.6%), pain; more toxicity with combo
Lyons et al. (2023) [18]	Well tolerated, no muscle spasms or serious AEs, performed under LA
Campana et al. (2022) [14]	33% ulceration (5% grade 3), mild pigmentation/suppuration
Campana et al. (2021) [13]	No serious AEs in any arm; immune AEs like pneumonitis noted
Borgognoni et al. (2020) [16]	Transient pain (27%), fever (52%), pigmentary changes (89.7%)
Petrelli et al. (2020) [21]	Generally mild; 1 treatment-related death (pre-existing lung disease)
Pirc et al. (2020) [22]	Adverse events not primary focus, safety assumed in modelling

In the large prospective InspECT registry analysis, Campana et al. (2022) observed grade 3 toxicity in only 5% of patients, with 49% (185/378) experiencing at least one adverse event within 30 days, most commonly ulceration (33%), hyperpigmentation (27%), suppuration (18%), and odour (12%) [14]. Systemic toxicity was negligible, and no serious treatment-related complications occurred. Campana et al. (2021) similarly demonstrated that combining ECT with pembrolizumab did not increase toxicity, with no serious adverse events in any treatment arm [13]. Lyons et al. (2023) also reported excellent tolerability using high-frequency electroporation (HF-EP), noting the absence of intra-procedural muscle contractions and no major complications. Approximately half of all procedures were safely performed under local anaesthesia, highlighting its suitability for elderly or frail patients [18].

Systematic reviews reinforced these findings, with Petrelli et al. (2020) reporting ECT as generally well tolerated across 27 studies, with pain, erythema, oedema, and ulceration being the most frequent adverse effects. One treatment-related death was reported in a patient with pre-existing pulmonary disease receiving intravenous bleomycin [21]. Ferioli et al. (2023) similarly described ulceration and necrosis as the main adverse effects, nearly all of which were mild-to-moderate and manageable with local care [20]. Ahmad et al. (2024) also reported minimal toxicity, even when ECT was combined with systemic or gene-based therapies [19]. In patients with in-transit melanoma, Campana et al. (2024) observed skin toxicity in 40%, including grade ≥3 ulceration in 15%, more frequently in those receiving combination treatment, yet overall tolerability remained high [15]. Borgognoni et al. (2020) noted no serious adverse events, with transient pain (27%), fever (52%), and pigmentary changes (89.7%) as the most common reactions [16]. Zdzienicki et al. (2024) and Pirc et al. (2020) did not explicitly report adverse-event data but raised no safety concerns [17,22].

Quality of Life Outcomes

QoL outcomes following ECT were variably assessed across studies, with the most comprehensive evaluation reported by Campana et al. (2022) using data from the prospective InspECT registry [14]. Patient-reported outcomes were measured with validated tools, including the EQ-5D-3L and EQ visual analogue scale (EQ-VAS). Overall QoL scores remained stable during follow-up, with no clinically significant decline observed. Patients achieving complete responses exhibited more favourable QoL trajectories, and baseline EQ-5D score emerged as the only independent predictor of treatment response (RR 14.76, p = 0.001) [14]. Higher baseline performance status and concomitant immunotherapy use were also associated with improved QoL throughout follow-up.

Pirc et al. (2020) incorporated EQ-5D-derived utility values into their health economic model and demonstrated that ECT generated gains in quality-adjusted life years (QALYs), particularly in patients with bleeding or ulcerated lesions, supporting its value in the palliative setting [22]. Lyons et al. (2023), although not employing formal QoL instruments, reported high patient satisfaction, minimal discomfort, and excellent tolerability with high-frequency electroporation (HF-EP), reinforcing its feasibility in outpatient and frail populations [18]. No formal QoL assessments were undertaken in the studies by Ahmad et al. (2024), Zdzienicki et al. (2024), Campana et al. (2021), Petrelli et al. (2020), Ferioli et al. (2023), or Borgognoni et al. (2020) [13,16,17,19-21].

Although the evidence for QoL improvements is narrow and largely reported by a registry study, several studies qualitatively described benefits such as treatment tolerability, outpatient delivery, and patient acceptability. Broader QoL assessments remain limited in the current ECT literature and remain an unmet need. 

Cost-Effectiveness

Cost-effectiveness was formally assessed in only one of the included studies. Pirc et al. (2020) conducted a discrete-time Markov model-based analysis comparing ECT with standard palliative care in patients with Stage IIIc/IV melanoma, using prospective clinical data from a Slovenian cohort [22]. Over a 10-year time horizon and from the perspective of the Slovenian healthcare system, ECT was associated with a mean gain of 0.29 QALYs at an incremental cost of €6568, resulting in a 30% probability of cost-effectiveness at a willingness-to-pay threshold of €20,000 per QALY (22). In a predefined subgroup of patients with bleeding lesions, QALY gains increased to 0.34, while incremental costs decreased to €4863, raising the probability of cost-effectiveness to 91%. Scenario analyses demonstrated further improvements in economic favourability with the removal of hospitalisation costs or a reduction in the cost of single-use electrodes, increasing cost-effectiveness probabilities to 58% and 64% respectively [22].

Although no other studies conducted formal economic analyses, several commented on ECT's cost-efficiency potential. Ahmad et al. (2024) and Lyons et al. (2023) both highlighted the feasibility of delivering ECT in outpatient settings under local anaesthesia, particularly with high-frequency electroporation (HF-EP), which may reduce procedural costs and improve access for frail patients. Lyons et al. also emphasised the benefit of avoiding general anaesthesia, thereby enhancing tolerability and potentially reducing perioperative resource utilisation [18]. Campana et al. (2022) and Borgognoni et al. (2020) noted that ECT’s favourable safety and repeatability profiles make it an attractive option for long-term local disease control, which could reduce the need for more invasive or costly interventions over time [14,16].

Overall, while current evidence supports the potential economic benefit of ECT within a single national health system, the external validity of these findings remains uncertain given that the principal Markov model was developed in one healthcare context. Robust, prospective cost-effectiveness studies are limited, and broader economic evaluations across diverse health systems are needed to fully define the financial impact of ECT and guide its integration into standard melanoma treatment pathways. 

Discussion

This systematic review suggests ECT is a safe, effective, and well-tolerated treatment for option for patients with stage III/IV melanoma, particularly those unsuitable for surgery or systemic therapy alone. Across the included studies, ECT consistently achieved high rates of local tumour control with minimal systemic toxicity, a favourable safety profile, and enhanced efficacy when combined with adjuvant immunotherapy. Collectively, these findings reinforce ECT as a valuable treatment option for those with malignant melanoma unsuitable for surgery or systemic chemotherapy.

Multiple studies reported strong local response rates with Campana et al. (2022), and Petrelli et al. (2020) reported ORRs of approximately 80% with CRRs of 47-48%, indicating sustained disease control over time [14,21]. Ahmad et al. (2024) observed ORRs up to 100% in selected subgroups, particularly when ECT was combined with immunotherapy. Long-term data from Borgognoni et al. (2020) confirmed that smaller lesions responded better to ECT, with CRR rates exceeding 59% [16,19]. Lyons et al. (2023) achieved CRR and ORRs of 85.7% and 100% using HF-EP, representing a potential refinement of standard ECT technology [18]. Evidence from Campana et al. (2024) confirms that ECT is repeatable, with response rates improving from 63% to 74% after retreatment [15]. These findings collectively underscore ECT’s capacity for durable local control and its applicability across a wide spectrum of melanoma presentations.

Observational data suggest that ET may have immunomodulatory effects that enhance the activity of immune checkpoint inhibitors. Both Ahmad et al. and Campana et al. (2021) reported increased CD8+ T-cell infiltration whilst reducing regulatory T-cell activity within the tumour microenvironment, which helps to potentiate checkpoint inhibitor efficacy and contribute to improved responses [13, 19, 23]. In Campana et al.’s matched cohort study, patients receiving pembrolizumab-ECT therapy achieved significantly higher CRRs and ORRs compared to pembrolizumab monotherapy (48.9% and 77.8%; 31.8% and 38.6%) and superior one-year LPFS and OS (86% vs 51%; 88% vs. 64%) [13]. Long-term survival benefits were further supported by Zdzienicki et al. (2024), who reported that the addition of immunotherapy post-ECT increased the five-year survival from 21% to 47% [17]. These findings suggest that ECT may not only provide effective local tumour control but also enhance survival outcomes when combined with immune checkpoint inhibitors. However, Ahmad et al. noted that CRR rates decreased when the tumour was irradiated before ECT, warranting further investigation to evaluate the relationship between radiotherapy and ECT [19]. Ahmad et al. (2024) also highlighted the potential of calcium chloride electroporation (Ca-EP) to maintain treatment efficacy while avoiding the chemotherapy-associated systemic toxicity [19]. These findings were observed in an RCT comparing the efficacy of Ca-EP with bleomycin-based EC,T which showed a non-significant difference in CRRs and ORRs (p = 0.30 for ORR, p = 0.45 for CRR), with Ca-EP being associated with fewer adverse events [24]. These findings suggest that Ca-EP is a safe and well-tolerated alternative with an improved side effect profile; however, further large-scale trials are needed to validate these results and define optimal electroporation protocols and clinical indications. 

Several studies also support the technical flexibility of ECT, with Petrelli et al. observing no significant difference in complete response between intravenous and intratumoural bleomycin administration, while tumour size did not significantly modify response [21]. The high CRR observed with HF-EP by Lyons et al., alongside its excellent tolerability, highlights this as a potential advancement in ECT delivery [18]. Moreover, across several studies, ECT was endorsed as a viable option for elderly or comorbid patients due to its safety and minimal systemic toxicity. 

Safety outcomes across all studies reaffirm ECT’s excellent tolerability, with adverse events primarily localised and transient-most commonly pain, erythema, oedema, and ulceration. Campana et al. (2022) observed grade 3 toxicity in only 5% of patients, and no meaningful increase in toxicity was identified when ECT was combined with immunotherapy (Campana et al., 2021) [13, 14]. A single treatment-related fatality was reported by Petrelli et al. in a patient with underlying lung disease treated with intravenous bleomycin, underscoring the need for careful patient selection [21]. From an economic perspective, the sole Markov model (Pirc et al., 2020) demonstrated gains in QALYs at acceptable incremental costs, especially for bleeding lesions [22]. Additional studies highlighted cost efficiencies from outpatient delivery and avoidance of general anaesthesia [18, 19]. However, as these findings are derived from a model developed within a single national health system, their broader generalisability remains uncertain, and further economic analyses across diverse healthcare settings are needed.

Despite these results, several limitations must be acknowledged. The evidence base is predominantly observational with marked heterogeneity in study design, patient selection, lesion characteristics, outcome reporting, and follow-up duration. This limited direct comparisons and precluded a meta-analysis. Reporting of lesion-based versus patient-based outcomes was inconsistent, and QoL and economic evaluations were sparse. Methodologically, this review relied on single-reviewer screening and data extraction, and no formal risk-of-bias assessment was performed, introducing potential for selection and interpretation bias. While efforts were made to apply inclusion and exclusion criteria rigorously, the absence of a second independent reviewer may have affected the objectivity and reproducibility of the review process. Taken together, these limitations highlight the need for prospective, standardised studies with robust methodology, systematic outcome reporting, and adequately powered evaluations of ECT alone and in combination with immunotherapy to further assess the role of ECT in the treatment of malignant melanoma. 

## Conclusions

This review confirms that ECT is a clinically safe, effective and well-tolerated treatment for melanoma, with high local tumour control rates and minimal toxicity. Its synergistic potential with immunotherapy, favourable QoL outcomes, and selective cost-effectiveness make it a compelling treatment option for melanoma. Future research should prioritise high-quality randomised control trials with standardised treatment and follow up protocols and further exploration of the effect of adjuvant immunotherapy with ECT is needed to confirm these findings and guide clinical adoption. 

## Appendices

Appendix A

**Table 4:** Search strategy and results

Search Line	Search Strategy (MEDLINE)	Results
1	Electrochemotherapy/	852
2	electrochemotherapy.mp.	1149
3	1 or 2	1149
4	exp Skin Neoplasms/	206556
5	skin cancer*.mp.	26406
6	skin neoplasm*.mp.	142943
7	dermatological cancer*.mp.	16
8	dermatological neoplasm*.mp.	6
9	Carcinoma, Basal Cell/	19263
10	Carcinoma, Squamous Cell/	145143
11	Melanoma/	101493
12	(basal cell carcinoma* or squamous cell carcinoma* or melanoma*).mp.	264903
13	4 or 5 or 6 or 7 or 8 or 9 or 10 or 11 or 12	413427
14	3 and 13	398
15	limit 14 to (english language and yr="2005 -Current")	341

Search Line	Search Strategy (EMBASE)	Results
1	electrochemotherapy/	1872
2	electrochemotherapy.mp	2074
3	1 or 2	2074
4	exp skin cancer/	171548
5	(skin cancer* or skin neoplasm*).mp.	68378
6	dermatological cancer*.mp.	36
7	dermatological neoplasm*.mp.	12
8	basal cell carcinoma/	33852
9	squamous cell carcinoma/	140209
10	melanoma/	172545
11	(basal cell carcinoma* or squamous cell carcinoma* or melanoma*).mp.	527763
12	5 or 6 or 7 or 8 or 9 or 10 or 11	558132
13	3 and 12	794
14	limit 13 to (English language and yr="2005 -Current")	712

Appendix B

**Table 5:** Included study characteristics

Study	Study design	Sample size	Treatment protocols	Key clinical outcomes	Safety outcomes / adverse effects	Other clinical outcomes
Campana et al. (2024)	Multicenter retrospective cohort study	22 centers, 452 patients with limb-only in-transit melanoma	ECT with intravenous bleomycin (96%), mostly hexagonal electrode (85%), 32% first-line ECT, 36% with concurrent treatment, 32% salvage ECT, 55% underwent retreatment	CR: 24% (initial), 39% (best); PR: 39% (initial), 35% (best); ORR: 63% (initial), 74% (best)	40% any-grade skin toxicity; 15% grade ≥3 ulceration; more adverse effects with combination strategies	Median local PFS: 2.75 years; OS: 5.7 years (CR: 6.4 vs non-CR: 5.2); 55% local recurrence
Ahmad et al. (2024)	Systematic review (51 studies incl. case reports, trials, reviews; PRISMA-guided)	51 included studies (14 on Stage III ECT; multiple cohorts across studies)	ECT primarily with bleomycin (IV or intralesional), ESOPE protocols used; some trials with cisplatin, IL-12 plasmid, immunotherapy (e.g. pembrolizumab, ipilimumab), calcium electroporation, or gene delivery.	Stage III: CR 75–100%, OR 53–100%; Stage III/IV: CR 15–74%, ORR 40–96%; ECT + ICI (pembrolizumab): ORR up to 77.8%	Generally low toxicity; radiotherapy before ECT negatively correlated with response. IL-12 electroporation inferior to ECT.	ECT + pembrolizumab improved OS (RR 2.02, p=0.046); some patients remained recurrence-free for >2 years; median OS not reached in several trials.
Zdzienicki et al. (2024)	Retrospective cohort study (2 Polish centers, 2010–2023)	88 patients with unresectable locoregional melanoma	ECT with IV bleomycin (15,000 IU/m²), Cliniporator device, hexagonal electrode; 11 patients received repeat ECT; ~70% received systemic therapy (chemo, ICI, BRAF/MEK)	CR: 45%, PR: 35%, ORR: ~80%	Not quantified; subjective lesion assessment a limitation; adverse effects not reported	OS: 1yr: 70%, 3yr: 51%, 5yr: 28.5%; Median OS: 37 mo; PFS: Median 5 mo, 1yr: 28.8%, 3/5yr: 14.3%; Immunotherapy after ECT improved OS in CR patients (HR 0.41, p=0.014)
Ferioli et al. (2023)	Systematic review (18 studies; PRISMA-compliant)	529 patients, 2,987 skin metastases from melanoma	ECT with bleomycin (15 studies), cisplatin (2), or both (1); IV or intratumoral administration; varied electrode types; 1–6 ECT cycles; 4 studies used concurrent chemo/immunotherapy	Lesion-based: CR 9–92% (pooled 53.5%), ORR 33.3–100% (pooled 77.0%); Patient-based: CR 15.2–50% (pooled 35.7%), ORR 66.7–100% (pooled 80.6%)	Common: pain (24–92%), erythema; Severe: necrosis (up to 41.6%), ulceration; toxicity higher with hexagonal electrodes and combined therapy	1-year LC: 80%; 1-year OS: 67–86.2%; some studies reported PFS and melanoma-specific survival variably
Lyons et al. (2023)	Prospective case series (proof-of-concept)	42 melanoma lesions analysed; 21 lesions at 18-month follow-up	High-frequency electroporation with bleomycin (IV or intratumoral); ePORE® device with CUTIS probes; HF biphasic pulses; local/general/spinal anaesthesia	3-month: CR 85.7%, PR 14.3%, ORR 100%; 18-month: CR 100% (n=21 lesions)	Well tolerated; no muscle spasms; no major AEs reported; feasible under local anaesthesia	Survival not reported; 4 lesions progressed by 12 months; promising long-term CR in HF-EP-treated melanoma
Campana et al. (2022)	Prospective observational cohort (InspECT registry)	378 melanoma patients across 27 centres	ECT per ESOPE guidelines; IV bleomycin (87%) or intratumoral (13%); Cliniporator used; varied electrodes; 13% received retreatment; some received post-ECT checkpoint inhibitors	CR: 47%, ORR: 80%, SD: 13%, PD: 5%; 1-yr LPFS: 78%, 2-yr: 68%, 3-yr: 62%	33% post-treatment ulceration (5% G3), hyperpigmentation, suppuration; low systemic toxicity; toxicity unaffected by CPI use	HRQoL stable overall; early drop in EQ-5D recovered by 4 months; better QoL in CR and CPI patients; baseline EQ-5D was independent CR predictor
Campana et al. (2021)	Comparative retrospective cohort study (3 groups: ECT, pembrolizumab, combo)	130 patients (45 combo, 44 pembrolizumab only, 41 ECT only)	ECT per ESOPE; bleomycin (IV/intratumoral), various electrodes; pembrolizumab standard dose; ECT delivered within 3 months of immunotherapy in combo group	Combo: ORR 77.8% (CR 48.9%, PR 28.9%); ECT: ORR 80.5% (CR 43.9%); Pembrolizumab: ORR 38.6% (CR 31.8%)	No serious AEs; local toxicity (ulceration, hyperpigmentation); immune-related AEs (e.g., pneumonitis); no added toxicity with combination	1-yr local PFS: Combo 86%, Pembrolizumab 51%; 1-yr systemic PFS: Combo 64%, Pembrolizumab 39%; 1-yr OS: Combo 88%, Pembrolizumab 64%; 2-yr OS: Combo 70%, Pembrolizumab 43%
Borgognoni et al. (2020)	Prospective observational single-centre study	44 melanoma patients, 244 melanoma nodules	ECT per ESOPE; IV bleomycin (65 patients), intratumoral (3); Cliniporator device; local, regional, or general anaesthesia based on tumour site/size; up to 3 sessions	Per patient: ORR 88.6% (CR 59.1%, PR 29.5%); Per lesion: ORR 82% (CR 58.6%, PR 23.4%); <3 cm nodules: CR 61.3%; ≥3 cm: CR 31.8%	No serious AEs; minor events: local pain (12), fever (23), ulceration (8), delayed healing (3); 89.7% had pigmentary changes	No recurrence in CR lesions at 6-month follow-up; median follow-up: 14 months; 36/68 alive, 32 died of systemic disease
Petrelli et al. (2020)	Systematic review and meta-analysis (27 studies)	1161 patients, 5308 melanoma cutaneous metastases	ECT with bleomycin (IV in 78%, intratumoral in 52%); ESOPE-guided pulses; RECIST/WHO used in 81%; cisplatin used in 4 studies (excluded from analysis)	Pooled ORR: 77.6%, CR: 48%; intratumoral bleomycin ORR: 81.9%, IV bleomycin ORR: 69.2% (p=0.37)	Pain (36–100%), erythema/edema (3–100%), ulceration (0–26%, G3 up to 18%); 1 fatal respiratory AE; generally mild toxicity overall	1-yr LCR: 54–89%; 2-yr LCR: 72–87%; 1-yr OS: 67–89% (in 3 studies); retreatment used in 26% of studies
Pirc et al. (2020)	Cost-effectiveness analysis using Markov model	27 Slovenian patients with stage IIIC–IV melanoma (retrospective input)	ECT costs and outcomes modelled against standard treatments (surgery, RT, chemo, immunotherapy); data from national registry and literature	Not reported directly; model based on observed CR/PR data from cohort and literature inputs	Not primary outcome; cost and QALY-based analysis	ECT associated with 1.18 QALYs vs 0.84 QALYs (standard); dominant strategy (more effective, less costly); ECT lifetime cost: €12,626 vs €17,662
